# Mapping National Governance of AI for Health: Protocol for a Global Scoping Review

**DOI:** 10.2196/88970

**Published:** 2026-07-23

**Authors:** Minmin Wang, Richelle George, Yu Zhao, Rajeshwari Singh, Kanika Kalra, Shada AlSalamah, Sameer Pujari, Yinzi Jin, Minghui Ren, Alain Labrique

**Affiliations:** 1 China Center for Health Development Studies, Peking University, Beijing, Beijing, China; 2 Beijing Institute for Health Development, Peking University, Beijing, China; 3 National Health Commission Key Laboratory of Health System Reform and Governance, Peking University, Beijing, China; 4 Artificial Intelligence and Frontier Technologies Unit, Department of Data, Digital Health, Analytics and AI, World Health Organization, Geneva, Switzerland; 5 Department of Global Health, School of Public Health, Peking University, No. 38 Xueyuan Road, Haidian District, Beijing, 100191, China, +86-15001081989

**Keywords:** health governance, artificial intelligence, AI, AI for health, national policy, scoping review

## Abstract

**Background:**

AI is rapidly transforming health systems, expanding from diagnostic imaging and predictive analytics to large language model–enabled clinical decision support. However, significant governance challenges persist, including algorithmic bias, privacy risks, limited transparency, and inequities in access. Despite the proliferation of national AI strategies, global governance remains fragmented, and systematic evidence on how national policies address ethical, regulatory, and implementation requirements is limited. No comprehensive synthesis currently maps national governance approaches against established frameworks or documents or accounts for implementation realities across diverse contexts.

**Objective:**

This scoping review aims to (1) characterize national approaches to AI governance in health, (2) assess alignment with established governance frameworks, and (3) identify implementation challenges and enabling factors.

**Methods:**

Following the Arksey and O’Malley framework and PRISMA-ScR (Preferred Reporting Items for Systematic Review and Meta-Analyses Extension for Scoping Reviews) guidelines, we searched 6 databases and key gray literature repositories for sources published between January 2015 and April 2025. Eligible documents include national-level policies, empirical analyses, and official reports on AI governance in health. Data extraction is guided by a framework integrating World Health Organization AI ethics and governance guidance and the strategic priorities of the Global Initiative on AI for Health across 4 dimensions—ethics, regulation, implementation, and operations. Descriptive mapping, governance principle coding, thematic synthesis, and subgroup analyses will be conducted.

**Results:**

Our systematic search across 6 electronic databases identified 21,278 records: 3409 (16.0%) from PubMed, 5691 (26.7%) from Embase, 3661 (17.2%) from Web of Science, 334 (1.6%) from Latin American and Caribbean Health Sciences Literature, 568 (2.7%) from the China National Knowledge Infrastructure, and 7615 (35.8%) from the WanFang Database. After removing 968 (4.6%) duplicates in EndNote (version V.21; Clarivate), 2 researchers independently screened 20,310 (95.5%) titles and abstracts. From 21,278 database records and 972 gray literature items, 149 (0.7%) sources met the inclusion criteria. Quality assessment and full data extraction will be finalized by June 2026.

**Conclusions:**

This review protocol addresses a critical evidence gap by providing a comprehensive mapping of national AI governance policies in health against an established governance framework. The planned review will inform evidence-based, equitable, and context-specific governance frameworks essential for safe and trustworthy AI integration in health systems.

## Introduction

AI is increasingly being applied in health systems worldwide, with applications ranging from diagnostic imaging and predictive analytics to personalized treatment and health system management. The recent emergence of generative AI and large language models has expanded these applications to include conversational health agents and clinical decision support tools, with these models demonstrating the ability to pass medical licensing examinations at or near passing thresholds and to assist with clinical diagnosis. However, concerns remain regarding accuracy limitations and hallucinations [1]. Recent evidence demonstrates AI’s transformative potential, including substantial reductions in diagnostic errors through predictive analytics [2]*,* enhanced precision medicine through integration of multimodal patient data [3]*,* and rapid proliferation with more than 343 Food and Drug Administration (FDA)–cleared AI-enabled medical devices by 2021 [4]. Large-scale implementation studies have demonstrated AI’s potential to improve efficiency while maintaining or improving clinical outcomes in population-level screening programs [5]. These technologies hold considerable promise for improving quality of care and population health outcomes.

At the same time, the rapid development and deployment of AI raise complex challenges, including ethical risks, data privacy concerns, potential algorithmic bias, and unequal access to opportunities. Algorithmic bias has been documented across multiple health care applications, with AI potentially perpetuating societal biases that can lead to misdiagnoses and adverse outcomes for underrepresented patient groups [6,7]. Privacy concerns are particularly acute, with only 11% of American adults willing to share health data with technology companies [8]. Studies show that more than 60% of health care professionals express hesitation in adopting AI systems due to transparency and data security concerns [9]. To ensure that AI contributes safely, equitably, and ethically to health systems, national-level governance frameworks are essential for providing oversight, setting standards, and safeguarding rights. In this review, governance of AI for health refers to the laws, policies, regulations, ethical guidelines, institutional arrangements, and accountability mechanisms through which governments oversee the development, deployment, and use of AI within health systems.

At the national level, many countries have implemented policies and strategies to govern the use of AI in health care. As of 2021, more than 50 national strategic initiatives for trustworthy AI exist across Organisation for Economic Co-operation and Development (OECD) member and partner countries, with more than 930 policy initiatives reported across 71 jurisdictions by May 2023 [10]. These initiatives differ substantially in scope, structure, and implementation, reflecting variations in institutional capacity, regulatory philosophies, and national priorities. Policy landscape reviews reveal that most countries are at an “emerging stage” of AI in health maturity, with significant heterogeneity in governance approaches [11,12]. While global reports and individual country case studies exist, evidence specific to the governance of AI in health remains limited and fragmented.

Current evidence explicitly documents that global regulation of health AI across member states remains fragmented, with laws and policies lacking formal cohesion. Multiple reviews confirm the absence of internationally recognized governance mechanisms for AI in health care [13], with recent reviews calling for more research on prerequisites and infrastructure for appropriate AI policy within health systems [14]. This fragmentation has been identified as a major barrier to AI adoption, with concerns about low public confidence potentially hindering AI’s translation to practice [15].

Established governance frameworks from academic literature and international organizations converge on core principles, including fairness, transparency, accountability, safety, privacy, and equity [16-19]. These include the OECD AI Principles [19], the European Union Artificial Intelligence Act [20], the United Nations Educational, Scientific and Cultural Organization (UNESCO) Recommendation on the Ethics of Artificial Intelligence [21], and regional initiatives from low- and middle-income countries (LMICs), such as the African Union’s Continental AI Strategy [22]. The analytical framework used in this review is grounded in World Health Organization (WHO) guidance, including that developed in collaboration with the International Telecommunication Union (ITU) and the World Intellectual Property Organization (WIPO). It is selected for its explicit focus on AI governance in health contexts, including LMICs. Consolidated evidence is lacking on the extent to which these are reflected in national approaches, the areas in which significant policy gaps exist, and what challenges countries face in implementation. Without comprehensive synthesis, opportunities to identify best practices, share lessons across contexts, and develop harmonized approaches remain limited.

A scoping review of national policies for AI in health governance will provide a systematic and comprehensive overview of the global landscape. By identifying and mapping existing policies, the review will highlight emerging governance models, assess the extent to which they embody principles of good governance, and illuminate common challenges faced by countries. Expanding the evidence base in this area is crucial for supporting policymakers, health system leaders, and international organizations in designing effective and context-specific governance frameworks. Ultimately, this work will contribute to ensuring that AI is governed in a manner that strengthens health systems and improves health outcomes.

## Methods

### Overview

This scoping review follows the established methodological framework of Arksey and O’Malley [23], which includes the following 5 stages: (1) identifying the research question; (2) identifying relevant studies; (3) study selection; (4) charting the data; and (5) collating, summarizing, and reporting the results. We will follow the PRISMA-ScR (Preferred Reporting Items for Systematic Review and Meta-Analyses Extension for Scoping Reviews) format for the publication of findings [24]. The protocol of the review has been registered with the PROSPERO (CRD420251234461).

### Stage 1: Identifying Research Questions

This scoping review aims to provide a comprehensive overview of national-level policies related to the governance of AI in health to inform efforts to strengthen health systems and improve health outcomes. The review is guided by 3 primary research questions. First, it examines how countries are structuring AI governance for health in relation to policy scope, institutional arrangements, regulatory mechanisms, and stakeholder engagement. Second, it assesses the extent to which national policies reflect established governance frameworks for AI in health. Third, it explores the implementation realities of AI governance by identifying the challenges countries face and the enabling factors that have been reported.

### Stage 2: Identifying Relevant Studies

We searched the following electronic databases: MEDLINE (PubMed), Embase, Web of Science, Latin American and Caribbean Health Sciences Literature (LILACS), China National Knowledge Infrastructure, and WanFang Data from January 1, 2015, to April 1, 2025. The start date was selected to capture the emergence of AI policy development in health, while the end date reflects the planned completion of searches.

In addition to peer-reviewed literature, we systematically searched gray literature and official reports from key international organizations and policy databases, including the WHO Institutional Repository for Information Sharing (IRIS), OECD AI Policy Observatory, United Nations AI Interagency Working Group, and Association of Southeast Asian Nations (ASEAN) websites. In addition, stakeholder consultation will be used to identify potentially relevant policy documents that may not be captured through database or repository searches.

The search strategy combines terms related to AI, health, and governance. AI-related terms include “artificial intelligence,” “machine learning,” “deep learning,” “natural language processing,” “large language model,” “large multimodal model,” and “generative AI.” Governance-related terms include “governance,” “policy,” “regulation,” “legislation,” “ethics,” “framework,” “guideline,” “strategy,” and “oversight.” Health-related terms include “health,” “healthcare,” “medicine,” “medical,” “public health,” and “health system.” Search terms will be adapted to the syntax and indexing structure of each database, including MeSH, where applicable. The detailed literature search strategy is provided in our online repository (Table S1 in Multimedia Appendix 1).

Gray literature searches will be conducted using a structured and reproducible approach. First, targeted searches will be performed within selected international repositories and policy databases using combinations of AI, health, and governance terms. Second, official websites of national governments, ministries of health, ministries responsible for digital transformation or science and technology, national AI offices, and health regulatory agencies will be searched to identify relevant national policy instruments. Third, targeted web searches will be conducted using combinations of country names and key terms such as “AI health policy,” “artificial intelligence healthcare regulation,” “AI governance health,” “digital health AI strategy,” and “AI ethics health.” Search results will be screened for official policy documents, legislation, strategies, regulatory guidance, and government reports. For each gray literature source, the document title, issuing organization, country, publication date, URL or repository source, and date accessed will be recorded.

### Stage 3: Study Selection

The primary unit of analysis in this review is the national-level policy instrument. For the purposes of this review, national-level policy instruments are defined as documents issued, enacted, commissioned, or formally endorsed by a national government, national ministry, national public health authority, national digital health agency, national AI authority, national regulatory agency, or other national-level public authority. Eligible primary policy texts include national legislation, regulations, national AI strategies, national digital health strategies with AI components, national health AI strategies, action plans, ethical frameworks, implementation roadmaps, regulatory guidance, standards, official policy frameworks, and government reports that address the governance, regulation, oversight, implementation, or ethical use of AI in health, health care, medicine, public health, or health systems.

Peer-reviewed empirical studies, case studies, cross-sectional studies, qualitative studies, quantitative studies, mixed methods studies, and policy analyses are eligible when they identify, describe, compare, or analyze specific national-level policy instruments related to AI governance in health. Official reports from international organizations or policy repositories are also eligible when they provide country-specific information on national AI-in-health policies or help identify relevant national policy documents. These supporting sources will be used to locate, verify, contextualize, and interpret national policy instruments.

Documents will be included if they address AI governance in relation to health, health care, medicine, public health, health systems, digital health, health data, clinical care, health research, or health service delivery. Documents focused on AI governance in general will be included only if they contain explicit health-related content or provisions relevant to health. No restrictions will be applied by geographic region, country income level, or language. Documents published in languages other than English or Chinese will be translated using DeepL Translate (DeepL SE) to facilitate screening, data extraction, and analysis.

The review will exclude systematic reviews, meta-analyses, conference abstracts, bibliometric analyses, editorials, opinion pieces, and commentaries that do not identify or analyze specific national policy instruments. Theoretical papers that discuss AI governance concepts without reference to specific national policies will also be excluded. Studies or documents focused solely on global, regional, institutional, subnational, or private-sector AI governance will be excluded unless they meet the criteria described above for inclusion as supporting evidence or nationally endorsed policy material.

The review focused on primary national policy texts, defined as government-issued instruments that articulate a country’s policy vision, legal provisions, or regulatory standards for AI in health, consistent with WHO’s health systems governance framework [25]. Such instruments may include national legislation, strategies, action plans, regulatory guidance, and official government reports.

Titles and abstracts of the retrieved citations were screened independently by 3 reviewers to identify relevant studies. We resolved any disagreements by consensus. All references were collated, and duplicate entries were removed.

### Stage 4: Charting the Data

We developed an analytical framework (Table S2 in Multimedia Appendix 1) based on WHO’s Ethics and Governance of Artificial Intelligence for Health [26] and the Global Initiative on AI for Health (GI-AI4H) strategic priorities [16]. The GI-AI4H is an ITU-WHO-WIPO–led multistakeholder platform that sets strategic priorities for responsible AI in health, including developing technical standards and policy guidance, facilitating knowledge and data sharing, and supporting evidence-based decisions on the introduction of health AI solutions [16].

The framework integrates WHO guidance [26-29] into 4 strategic dimensions—ethics, regulation, implementation, and operations—encompassing 25 guiding principles for AI governance. These principles address topics including transparency, accountability, data protection, equity, workforce readiness, and regulatory oversight, among others.

Data will be extracted from each included source using a standardized data extraction form covering general information, governance content, and governance challenges and recommendations. General information will include publication year, country or regional focus, document type, and type of AI technology referenced. Governance content will be assessed against each of the 25 principles listed in Table S2 in Multimedia Appendix 1.

For governance content, each of the 25 principles will be coded using a 4-category coding framework: comprehensively addressed, partially addressed, indirectly addressed, or not addressed. A principle will be coded as comprehensively addressed when the policy explicitly discusses the principle and provides operational details, such as concrete provisions, responsible institutions, implementation mechanisms, regulatory requirements, monitoring procedures, or enforcement arrangements. A principle will be coded as partially addressed when the policy explicitly mentions or discusses the principle but provides limited detail on how it will be implemented, monitored, enforced, or assigned to responsible actors. A principle will be coded as indirectly addressed when the policy does not directly name the principle but refers to related concepts, objectives, or activities that are relevant to the principle. A principle will be coded as not addressed when the document contains no relevant reference to the principle or its underlying concept. This coding approach enables both quantitative analysis of policy coverage and qualitative assessment of governance approaches. Information on governance challenges and recommendations will include barriers to and enablers of implementation, gaps in existing policies, and proposed solutions to address governance challenges.

Two researchers will independently extract data from all included studies using the data extraction framework in Microsoft Excel. During the conduct of the full review, a pilot extraction exercise and a coding guide will be implemented to strengthen consistency and reproducibility. Interreviewer agreement will be assessed during the initial calibration phase to support methodological rigor and consistency. Any discrepancies will be resolved through discussion; if disagreements persist, a third researcher will be invited to deliberate.

### Stage 5: Collating, Summarizing, and Reporting the Results

We will conduct a descriptive analysis of AI governance policies by period, country or region, and specific governance elements within the 4 strategic dimensions of ethics, regulation, implementation, and operations. The analysis will examine temporal trends in policy development, the geographic distribution of policies by WHO region and income level, policy maturity and scope, and the extent to which the 25 governance principles are addressed. This will allow us to identify which governance principles are most and least commonly reflected in national AI-in-health policies.

Thematic analysis will be used to identify recurring themes related to policy challenges and recommendations, and findings will be synthesized narratively. Where feasible, the frequency of reported challenges will be documented and compared across regions. To enhance the interpretability and robustness of the findings, we will conduct sensitivity or subgroup analyses examining differences between high-income countries and LMICs, variation by policy type, such as legislation, strategy documents, and regulatory guidance, and the temporal evolution of governance approaches. Results will be presented through tables, figures, and narrative synthesis, and the PRISMA-ScR checklist will be applied to ensure comprehensive reporting.

## Results

### Literature Search and Screening Results

Our systematic search across 6 electronic databases identified 21,278 records: 3409 (16.0%) from PubMed, 5691 (26.7%) from Embase, 3661 (17.2%) from Web of Science, 334 (1.6%) from LILACS, 568 (2.7%) from the China National Knowledge Infrastructure, and 7615 (35.8%) from the WanFang Data. After removing 968 (4.6%) duplicates in EndNote (version 21; Clarivate), 2 researchers independently screened 20,310 (95.5%) titles and abstracts.

Gray literature searches identified 972 additional items from WHO IRIS, OECD AI Policy Observatory, and the United Nations AI Interagency Working Group.

**Figure 1.**
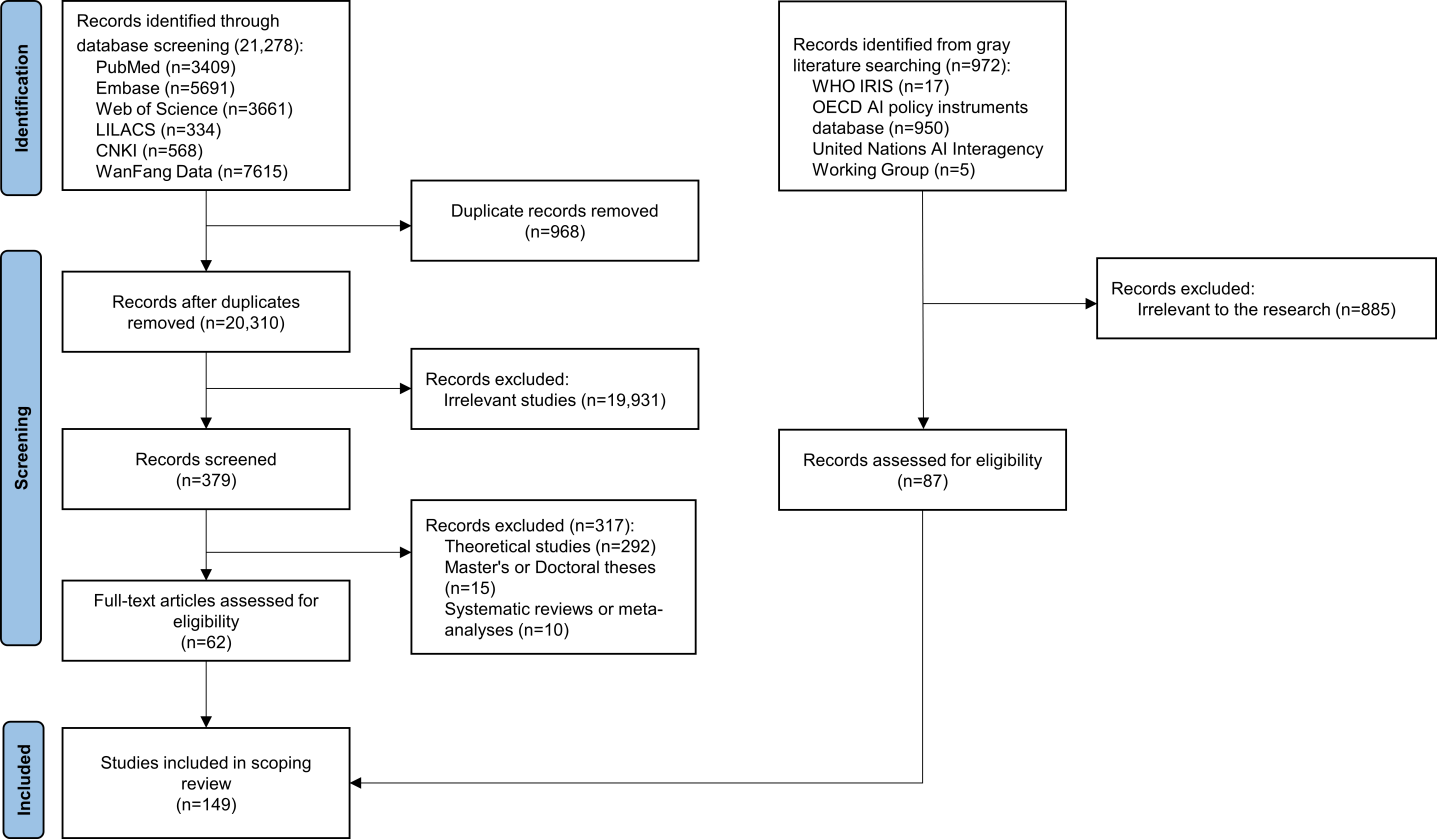
PRISMA (Preferred Reporting Items for Systematic Reviews and Meta-Analyses) flow diagram. CNKI: China National Knowledge Infrastructure; IRIS: Institutional Repository for Information Sharing; LILACS: Latin American and Caribbean Health Sciences Literature; OECD: Organisation for Economic Co-operation and Development; WHO: World Health Organization.

A total of 149 records met the inclusion criteria: 62 (41.6%) peer-reviewed articles and 87 (58.4%) comprising gray literature items and official reports. Figure 1 summarizes the identification, search, and screening process.

### Status of Review Completion

Quality and risk-of-bias assessment using the Agency for Healthcare Research and Quality (AHRQ) methodology checklist is planned for the empirical studies. The AHRQ methodology checklist contains 11 items and is rated based on the overall score. For each item, one score is awarded if the quality of the study meets the methodological standard. The quality assessment and data extraction of the included studies and policies will be finalized by June 2026.

## Discussion

The integration of AI into health systems represents a defining challenge for contemporary health governance. While AI holds transformative potential to improve diagnostics, efficiency, and health care access, realizing these benefits depends on robust governance frameworks [1,6-9]. Despite emerging international guidance on AI ethics and governance [16,19,21,26-29] and proliferating national strategies, the global landscape remains fragmented and poorly understood [11-14]. By synthesizing the existing landscape, this review will underscore the critical importance of understanding national AI governance in health as both a technical and political domain. Results from this scoping review could address a critical evidence gap by systematically mapping national governance approaches, assessing alignment with established governance frameworks, and documenting implementation realities across diverse contexts.

This review is expected to accelerate countries’ establishment of governance structures across 4 critical dimensions: regulation, ethics, implementation, and operations. By mapping approaches to AI for health governance frameworks, the review will provide concrete models for policymakers to adapt to national contexts. Particular attention will also be paid to equity considerations, including mechanisms that safeguard minority, indigenous, and disadvantaged populations, and approaches addressing resource disparities between high-income and low- and middle-income settings [21,30]. Recent evidence shows AI development remains concentrated in high-income nations, often overlooking needs in resource-constrained settings [30,31]. The analysis will examine cross-border coordination, regulatory harmonization, and knowledge and resource flows to resource-limited contexts, thereby informing high-level policy dialogue and diplomacy on international AI governance cooperation [32-34]. By situating national policies within the broader landscape of international AI governance norms, the review will also enhance international comparability and enable assessment of how global frameworks are being translated into national health-sector governance.

Beyond cataloging policies, this review will assess implementation realities—challenges countries face in translating governance frameworks into practice and enabling factors facilitating successful adoption. This focus is critical, as frameworks often fail to account for infrastructure limitations, capacity constraints, and contextual factors determining whether AI systems can be deployed safely and equitably [35-38]. Importantly, even well-designed governance frameworks may prove difficult to operationalize without accompanying implementation support, including context-specific incentive structures, dedicated institutional capacity, and sustained political commitment. By documenting barriers from inadequate infrastructure and data quality to workforce capacity and financial sustainability, the review will provide actionable insights for policymakers.

The review’s strengths include its comprehensive search strategy encompassing peer-reviewed and gray literature across multiple languages, capturing diverse governance approaches from countries at different AI maturity stages. The analytical framework, grounded in WHO guidance and the priorities of the GI-AI4H, provides a systematic assessment of policy alignment with established frameworks while identifying novel approaches. The multidisciplinary team brings expertise in AI ethics, health policy, global governance, and implementation science. Moreover, this review serves as a foundational resource for WHO’s normative guidance and contributes to evidence-informed dialogue within the GI-AI4H.

Several limitations warrant acknowledgment. First, the rapidly evolving field means that policies may emerge between search completion and publication. Second, the analysis relies on documented policies, which may not reflect implementation realities, informal mechanisms, or gaps between stated policy and practice. Countries with greater capacity may have more comprehensive documentation, potentially skewing evidence toward high-income settings. Although we will search major international and regional policy repositories and supplement these searches through stakeholder consultation, some national policy documents may remain difficult to identify if they are not indexed in commonly used repositories or are available only through country-specific ministry websites. Third, translation tools may imperfectly capture linguistic nuances and context-specific concepts. Finally, the framework-based approach may miss emergent governance innovations outside established WHO guidance categories.

Despite these limitations, the planned review is expected to provide a comprehensive synthesis of national AI governance policies in health, drawing on 149 sources from peer-reviewed and gray literature in multiple languages. By systematically mapping existing policy instruments, the review aims to describe how national AI health governance is currently structured, identify areas where governance principles are more or less clearly addressed, and highlight potential policy gaps, implementation challenges, and enabling factors. As AI technologies continue to advance, evidence-informed and context-sensitive governance frameworks are increasingly important. This review is intended to support more nuanced dialogue in this rapidly evolving field and to inform future efforts to responsibly harness AI’s potential to strengthen health systems while safeguarding equity, ethics, and human rights.

## Supplementary material

10.2196/88970 Multimedia Appendix 1 Searching strategy for the literature review and analytical framework for governance of artificial intelligence for health.

## Abbreviations

AHRQ: Agency for Healthcare Research and Quality
ASEAN: Association of Southeast Asian Nations
FDA: Food and Drug Administration
GI-AI4H: Global Initiative on AI for Health
IRIS: Institutional Repository for Information Sharing
ITU: International Telecommunication Union
LILACS: Latin American and Caribbean Health Sciences Literature
LMIC: low- and middle-income country
OECD: Organisation for Economic Co-operation and Development
PRISMA-ScR: Preferred Reporting Items for Systematic Review and Meta-Analyses Extension for Scoping Reviews
UNESCO: United Nations Educational, Scientific and Cultural Organization
WHO: World Health Organization
WIPO: World Intellectual Property Organization
