# Supplementary material for: Mapping National Governance of AI for Health: Protocol for a Global Scoping Review
Source: JMIR Res Protoc. 2026 Jul 23;15:e88970. doi: 10.2196/88970 (PMC13394865; doi:10.2196/88970)
Supplement: Multimedia Appendix 1 [file resprot-v15-e88970-s001.docx]

**Mapping National Governance of AI for Health: A Global Scoping Review**

Supplemental Table 1. Searching strategy for the literature review

**PubMed**

| **Search** | **Query** | **Result** |
| --- | --- | --- |
| #1 | TI ("AI" OR "artificial intelligence" OR "LMM" OR "Large Multi-modal Models" OR "generative AI" OR "Natural Language Processing" OR "machine-learning") | 1976 |
| #2 | AB ("AI" OR "artificial intelligence" OR "LMM" OR "Large Multi-modal Models" OR "generative AI" OR "Natural Language Processing" OR "machine-learning") | 19,895 |
| #3 | SU ("AI" OR "artificial intelligence" OR "LMM" OR "Large Multi-modal Models" OR "generative AI" OR "Natural Language Processing" OR "machine-learning") | 58,631 |
| #4 | TI ("Governance" OR "policy" OR "regulate" OR "ethics") | 2457 |
| #5 | AB ("Governance" OR "policy" OR "regulate" OR "ethics") | 21,215 |
| #6 | SU ("Governance" OR "policy" OR "regulate" OR "ethics") | 109,718 |
| #7 | 2015/01/01:2025/04/01[Date - Publication] | 13,965,932 |
| #8 | #1 OR #2 OR #3 | 78,276 |
| #9 | #4 OR #5 OR #6 | 130,312 |
| #10 | #8 AND #9 | 4241 |
| #11 | #7 AND #10 | 3409 |

**Web of Science**

| **Search** | **Query** | **Result** |
| --- | --- | --- |
| #1 | TI=("AI" OR "artificial intelligence" OR "LMM" OR "Large Multi-modal Models" OR "generative AI" OR "Natural Language Processing" OR "machine-learning") | 461,825 |
| #2 | TI=("Governance" OR "policy" OR "regulate" OR "ethics") | 1,281,292 |
| #3 | DOP=(2015-01-01/2025-04-01) | 79,151,272 |
| #4 | #1 AND #2 | 3780 |
| #5 | #3 AND #4 | 3661 |

**Embase**

| **Search** | **Query** | **Result** |
| --- | --- | --- |
| #1 | (ai:ab,ti OR 'artificial intelligence':ab,ti OR lmm:ab,ti OR 'large multi-modal models':ab,ti OR 'generative artificial intelligence':ab,ti OR 'natural language processing':ab,ti OR 'machine learning':ab,ti) | 285,490 |
| #2 | (governance:ab,ti OR policy:ab,ti OR regulate:ab,ti OR ethics:ab,ti) | 917,303 |
| #3 | #1 AND #2 | 6569 |
| #4 | #3 AND [01-01-2015]/sd NOT [01-04-2025]/sd | 5691 |

**LILACS**

| **Search** | **Query** | **Result** |
| --- | --- | --- |
| #1 | Title, abstract, subject =(AI) OR (artificial intelligence) OR (LMM) OR (Large Multi-modal Models) OR (generative AI) OR (Natural Language Processing) OR (machine-learning) | 4770 |
| #2 | Title, abstract, subject = (Governance) OR (regulate) OR (ethics) OR (policy) | 53,321 |
| #3 | #1 AND #2 | 390 |
| #4 | limit 3 to year: [2015-01-01 TO 2025-04-1] | 334 |

WanFang Database

(人工智能 OR 大模型 OR 生成式人工智能) AND (政策 OR 规定 OR 条例) AND (医疗 OR 卫生健康) ,year＝[2015-01-01 TO 2025-04-1] ,N=7615

CNKI

('人工智能' + '大模型' + '生成式人工智能') * ('政策' + '规定' + '条例') * ('医疗' + '卫生健康') ,year＝[2015-01-01 TO 2025-04-1] , N=568

Supplemental Table 2. Analytical framework for governance of AI for health

| **Strategic Priorities** | **Detailed guiding principles** |  |
| --- | --- | --- |
|  |  |  |
| Ethics | - Communities should have the right to oversee and manage their data usage. - Decision-making for introducing AI systems should be democratized. - Ethical guidelines, aligned with international data protection principles, should be established to prevent data bias. - AI technologies should promote health equity. - Global governance should ensure that AI development aligns with ethical, human rights, and legal obligations. - Marginalized groups should receive special attention. |  |
| Regulation | - Governments should establish comprehensive legal and regulatory frameworks. - Transparency in regulatory procedures and interoperability should be enhanced. - Online health services should be regulated to ensure patient safety and integration with formal healthcare systems. - Clear regulatory standards should be introduced. - Governments should collaborate with the private sector to regulate AI technologies while enhancing their own regulatory capacity. - The scope of private sector involvement and government responsibilities should be clearly defined. - Governments should establish independent data protection authorities. |  |
| Implementation | - Explore alternative incentives beyond intellectual property rights to promote research and development. - Validate AI system performance through prospective randomized trials. - Prioritize transparency in public-private partnerships. - Conduct transparent and inclusive impact assessments to establish ethical and legal standards for AI procurement. - Pursue collaboration with non-governmental and community organizations. - Assign dedicated teams to perform objective peer reviews of software and system implementations. |  |
| Operations | - Ensure transparency and compliance in data hubs for both public and private uses to maximize public benefit. - Allow governments, research institutions, and universities to retain ownership of AI development outcomes. - Ensure AI technologies are transparent while respecting intellectual property rights. - Require entities to disclose data usage scopes transparently. - Disclose AI applications in healthcare, including investments, partnerships, and risks. - Integrate incentives for developers into precertification programs and strengthen market surveillance. |  |
